# Adequacy of electronic and in-person referrals to endocrinology: a prospective study of primary care and hospital consultations

**DOI:** 10.3389/fendo.2026.1864526

**Published:** 2026-07-24

**Authors:** Juan J. Díez, Macarena Alpañés, Pedro Iglesias, Raquel Mateo

**Affiliations:** 1 Department of Endocrinology, Hospital Universitario Puerta de Hierro Majadahonda, Majadahonda, Spain; 2 Instituto de Investigación Sanitaria Puerta de Hierro Segovia de Arana, Majadahonda, Spain; 3 Department of Medicine, Universidad Autónoma de Madrid, Madrid, Spain

**Keywords:** electronic consultation, endocrinology, health services organization, primary care, prioritization, referral adequacy

## Abstract

**Purpose:**

To assess the adequacy of electronic consultations (e-consults) and in-person referrals to an endocrinology department, to describe patterns and causes of inadequacy, and identify factors associated with inadequacy across different referral pathways.

**Methods:**

All e-consults and in-person referrals received by the Department of Endocrinology of a tertiary hospital were prospectively recorded over a 52-working-day period. Consultations were classified as primary care e-consults, primary care in-person referrals, or hospital specialist in-person referrals. Each consultation was evaluated for adequacy and, when inadequate, categorized according to clinical or administrative causes. Priority assignment of primary care in-person referrals was also assessed. Univariable and multivariable logistic regression analyses were performed separately for primary care and hospital in-person referrals to identify factors associated with referral inadequacy.

**Results:**

A total of 1,106 consultations were analyzed (e-consults, 187; primary care in-person referrals, 447; hospital in-person referrals, 472). Overall adequacy was 61.8%, with higher adequacy in primary care e-consults (77.5%) than in primary care (63.1%) and hospital in-person referrals (54.4%). Administrative causes predominated among inadequate e-consults, whereas hospital referrals showed both clinical and administrative causes. Among primary care in-person referrals, 46.3% were assigned priority, of which 58.5% were considered inappropriately prioritized. The association between age and adequacy did not follow a linear pattern, with the 41–64 age group showing the highest proportion of inadequacy in both primary care and hospital referrals. Referral origin outside the hospital catchment area was associated with higher inadequacy in primary care referrals.

**Conclusion:**

Referral inadequacy to endocrinology services is common and varies by consultation modality, diagnostic category, and organizational factors. Targeted strategies to optimize referral pathways and prioritization may improve efficiency and access to specialist endocrine care.

## Introduction

Endocrine and metabolic disorders represent a substantial proportion of chronic diseases encountered in both primary care and hospital-based specialist settings, contributing to an increasing demand for endocrinology services (1–5). This trend is supported by epidemiological data. The 11th edition of the International Diabetes Federation (IDF) Diabetes Atlas reported a global prevalence of 11.11% in 2024 (589 million adults), with projections indicating an increase to 12.96% (853 million) by 2050 (6). In Spain, projections indicate that the number of adults with diabetes will rise from 2.8 million in 2011 to an estimated 4.9 million in 2050 (7). Furthermore, the prevalence of overweight and obesity has also increased steadily, further contributing to the growing burden of endocrine and metabolic disease (8).

Traditionally, clinical referrals have relied on in-person consultations, whereby primary care physicians or other specialists seek expert assessment and management for complex cases that require specialized knowledge (9). In recent years, telemedicine-based communication tools have emerged as effective means to streamline collaboration between primary and secondary care. Electronic consultations (e-consults) allow primary care clinicians to submit patient-specific clinical queries to specialist teams asynchronously, enabling timely recommendations without requiring a face-to-face visit (10, 11). Evidence increasingly supports the efficiency, feasibility, and acceptability of e-consult systems in endocrinology, highlighting reduced wait times, improved prioritization of referrals, and high provider satisfaction (11–13).

In our healthcare setting, e-consultation is a telematic system that enables clinical interconsultation between primary care physicians and hospital-based specialists without the physical presence of the patient. It allows the secure exchange of clinical information through a dedicated software platform (Sistema Integrado de Peticiones, SIPE), which has been implemented in our hospital since 2020 (14). Therefore, at the time of the study, both primary care physicians and specialists were familiar with its routine use. The e-consultation model in our setting is unidirectional: the primary care physician submits a clinical query to the specialist, who provides a single response based on the available information. This interaction does not typically involve iterative dialogue. Clinical decision-making remains the responsibility of the primary care physician, who integrates the specialist’s recommendations into patient management. On the other side, e-consults may be requested for both newly referred patients and those already under specialist care, including shared-care models between primary and hospital care. In our public healthcare system, e-consultations are not associated with financial incentives or reimbursement. Their use is based exclusively on clinical judgment.

The adequacy of an in-person referral is a key determinant of efficient specialist care. Appropriate referrals should concern clinically relevant problems that cannot be adequately resolved through telematic communication, must include sufficient clinical information, should reflect suitable urgency, and should justify specialist involvement based on patient complexity (15). Inadequate referrals may arise when cases fall outside the consulted specialty’s scope, reflect non-clinical motives, involve poorly defined questions, or lack essential clinical data (16, 17). Process-related deficiencies—such as incomplete information, unclear communication, or exclusion of involved professionals—also contribute to suboptimal referral quality (17).

Given the rising prevalence and chronicity of endocrine and metabolic disorders, the increasing number of referrals to endocrinology services is expected to continue (1–5). Inadequate referrals can exacerbate system inefficiencies—prolonging waiting lists, delaying care for patients requiring urgent evaluation, increasing clinician workload, and decreasing satisfaction among both patients and healthcare professionals (18). Understanding the prevalence, characteristics, and determinants of inadequate referrals is therefore crucial for implementing targeted interventions that reduce unwarranted variability and improve referral quality.

The present study aims to evaluate the adequacy of all types of consultations (both electronic and in-person) submitted to the endocrinology department, to characterize the patterns and causes of inadequacy, and to assess variability across referring services. Identifying modifiable factors underlying inadequate referrals will support the development of corrective strategies to enhance communication between care levels, rationalize specialist demand, and improve overall clinical efficiency.

## Methods

### Study setting and classification of referrals

All in-person referrals and electronic consultations (e-consults) received by the Department of Endocrinology and Nutrition at Hospital Universitario Puerta de Hierro Majadahonda (HUPHM) were prospectively recorded over a 52-working-day period, from 12 January to 24 March 2026. Each referral was categorized into one of three groups: primary care e-consults, primary care in-person referrals, and hospital specialist in-person referrals. Hospital-based referrals were additionally classified according to whether they originated from medical or surgical specialties. Referrals from primary care were stratified by type of center, i.e., health district reference centers (large centers supervising multiple satellite clinics), local consulting clinics (small primary care facilities) or outside the hospital’s catchment area. The unit of analysis was the individual referral (e-consult or in-person referral). Each referral was evaluated independently, and no attempt was made to link multiple referrals to the same patient or to determine whether different referrals addressed the same clinical question.

**Figure 1:**
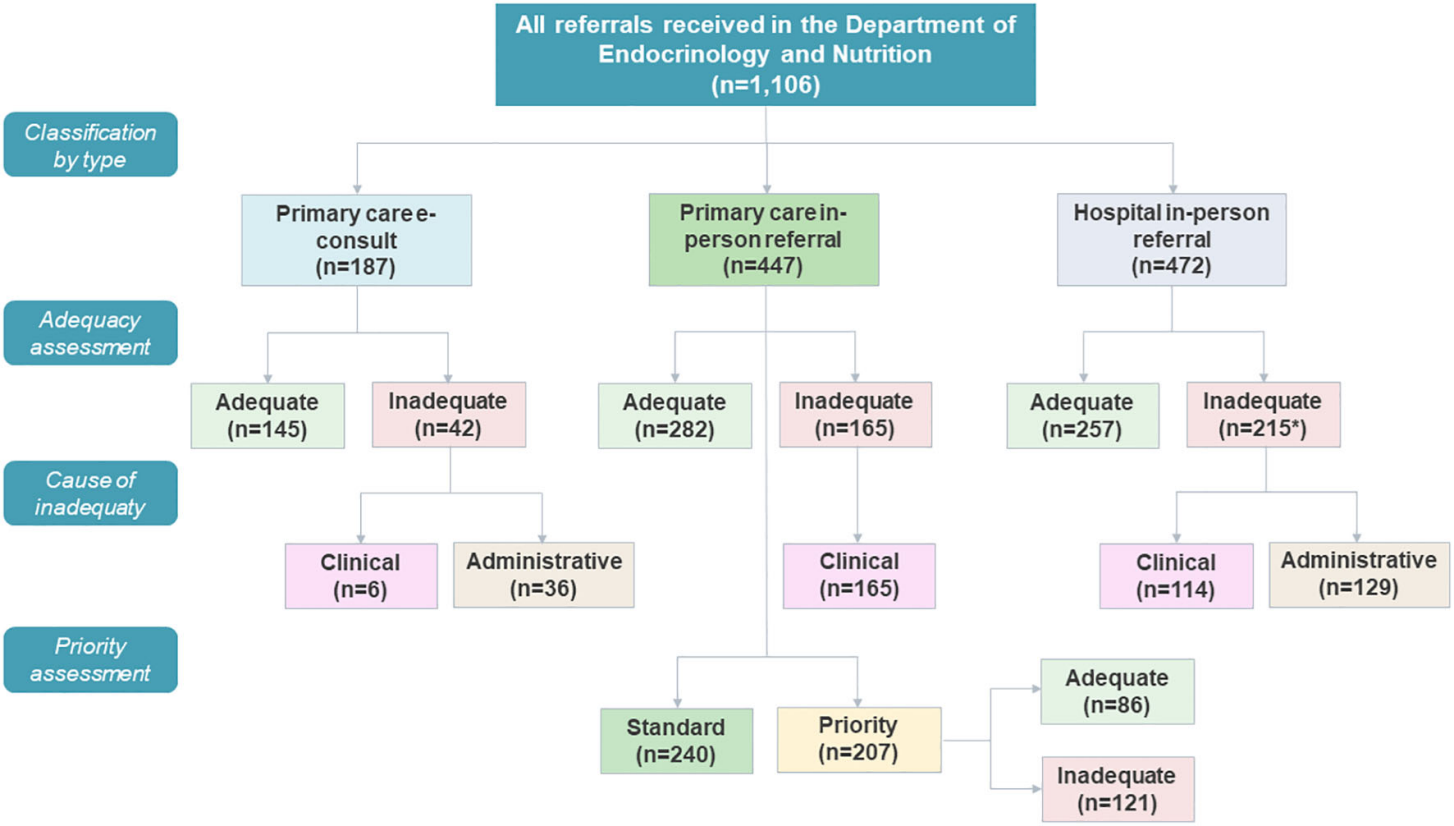
Flowchart of referral classification and adequacy assessment. The diagram illustrates the inclusion of all referrals during the study period and their categorization into e-consults, primary care in-person referrals, and hospital specialist referrals, as well as the subsequent assessment of adequacy and, when applicable, priority. *In hospital specialist referrals, of the 215 inadequate cases, 86 were classified as clinically inadequate, 101 as administratively inadequate, and 28 as having both causes. Accordingly, the totals displayed for clinical (86 + 28) and administrative (101 + 28) causes are not mutually exclusive.

For primary care in-person referrals, the priority level assigned by the referring physician was documented as standard or preferential. Standard referrals followed routine scheduling and were assigned the next available appointment according to the waiting list without specialist review. Preferential referrals were used when the referring physician considered that earlier assessment was required; in these cases, the referral was reviewed by the receiving specialist to determine the need for expedited evaluation. Priority classification was not applied to hospital-based referrals, all of which were considered standard. A flowchart summarizing the classification and assessment process of referrals is provided in **Figure 1**.

For every in-person referral and e-consult, patient sex and age were recorded. Referrals and e-consults were grouped into the following nosological groups corresponding to the specialty of endocrinology and nutrition: thyroid dysfunction, nodular thyroid disease (NTD), diabetes, obesity, dyslipidemia, disorders of mineral metabolism, adrenal disease, nutritional disorders (excluding obesity), and other endocrine conditions not fitting the previous classifications. In all cases, each referral or e-consult was evaluated for adequacy. Inadequate referrals were further classified as having clinical or administrative causes.

### Outcome definition

Clinical adequacy was defined according to a set of predefined criteria developed through a structured, iterative process. Initially, these criteria were drafted by members of the Department of Endocrinology and Nutrition based on routine clinical practice and common referral scenarios. The proposed criteria were subsequently presented during a cycle of five structured educational sessions delivered by the department to primary care physicians within the hospital catchment area in November 2023. During this educational activity, attending primary care physicians were encouraged to provide feedback, ask questions, and suggest modifications. Following this interactive process, the criteria were revised accordingly and subsequently discussed, refined, and formally approved by all members of the Department of Endocrinology and Nutrition (n=12 physicians; 4 primarily dedicated to clinical nutrition and 8 to endocrinology). No external independent panel participated in this process. Adequacy was defined dichotomously (adequate vs. inadequate) based exclusively on these predefined criteria.

### Assessment procedure

Assessment of referral adequacy was performed by four endocrinologists from the department (the authors of this study). All evaluators applied the same predefined criteria, previously agreed upon in a formal departmental clinical session. Explicit use of standardized criteria was required in all cases. In situations where uncertainty arose regarding classification, the case was jointly reviewed and a final decision was reached by consensus between two or more evaluators. No formal inter-rater agreement analysis (e.g., kappa statistics) was conducted. Importantly, adequacy assessment was conducted retrospectively and was fully independent from clinical decision-making. The evaluators did not influence the management of referrals, including responses to e-consultations or decisions regarding face-to-face appointments. Clinicians responsible for patient care were unaware of the adequacy classification assigned in this study. In the case of specialist-to-specialist referrals, the assessment was limited to the adequacy of the referral to the Department of Endocrinology and Nutrition. Previous referral steps within the healthcare pathway (e.g., initial referral from primary care to another specialty) were not evaluated.

### Criteria for assessing adequacy in e-consults

#### Clinical inadequacy

Adequacy was assessed using a binary classification (adequate vs. inadequate), based on the extent to which the e-consult fulfilled its intended purpose within our healthcare system. In our setting, e-consultation is specifically designed as a tool for resolving clinical queries between primary care physicians and hospital-based specialists. Therefore, adequacy assessment focused exclusively on the formulation, relevance, and clinical usefulness of the question posed, rather than on patient outcomes, disease severity, or the need for subsequent healthcare interventions.

An e-consultation was considered clinically inadequate when it did not allow an appropriate clinical response to be provided. This included (1): insufficient clinical information, where relevant data were lacking to support a reasoned response, and (2) non-pertinent e-consult, defined as the absence of a clear clinical question or a query that did not fall within the scope of endocrinology and nutrition (**Supplementary Table 1**; **Supplementary Material**). Importantly, this classification was independent of whether the patient ultimately required a face-to-face visit, hospital admission, telephone consultation, or no further action.

#### Administrative inadequacy

Administrative inadequacy was defined according to the criteria of the Gerencia Asistencial de Atención Primaria de la Comunidad de Madrid (Primary Care Management of the Community of Madrid) (19), which stipulate that e-consults must be exclusively clinical in purpose. Therefore, e-consults were classified as administratively inadequate when their primary purpose was non-clinical, including appointment management, referral modifications, or administrative procedures unrelated to patient care. (**Supplementary Table 1**, **Supplementary Material**).

### Criteria for assessing adequacy in in-person referrals

#### Clinical inadequacy in in-person referrals

Clinical inadequacy was defined according to three predefined categories, consistently applied to referrals from both primary care and hospital-based services (**Supplementary Table 1**; **Supplementary Material**): (i) insufficient clinical information, when the referral lacked the necessary detail to allow an appropriate assessment; (ii) condition manageable within primary care, defined according to diagnostic-specific criteria (**Supplementary Table 2**; **Supplementary Material**); and (iii) condition not related to Endocrinology and Nutrition, when the clinical problem fell outside the scope of the specialty. These categories were designed to be exhaustive, and all clinically inadequate referrals were assigned to one of them.

#### Administrative inadequacy in in-person referrals

Administrative inadequacy was not considered applicable to referrals originating from primary care, as referral pathways between primary and specialized care are structurally embedded within the organizational framework of the Spanish National Health System. For hospital-based referrals, administrative inadequacy was assigned when the patient did not reside within the hospital’s designated catchment area, and the referral reason was not directly related to the condition for which the patient was being followed in the referring specialty (**Supplementary Table 1**; **Supplementary Material**).

#### Assessment of priority adequacy in primary care referrals

Priority adequacy for primary care in-person referrals was evaluated based on the clinical situation at the time of referral, considering urgency, diagnostic or therapeutic needs requiring hospital-based resources, and appropriateness of specialist involvement. Clinical complexity was interpreted in context, as it does not necessarily determine urgency: a complex patient may present with a non-urgent issue, while a less complex patient may require priority evaluation due to acute or high-risk features. Since no institution-wide standardized priority document exists, decisions were made according to the following systematically applied principles: (i) presence of alarm signs or symptoms, or reasonable suspicion of severe or time-sensitive disease, (ii) risk of significant clinical deterioration if specialist evaluation were delayed, (iii) need for immediate diagnostic or therapeutic intervention unavailable in primary care, (iv) suspicion of endocrine neoplasia or serious endocrine complications, and (v) requirement for specialist input for time-dependent clinical decisions. Appropriateness of specialist involvement was defined as whether the referral was directed to the specialty of endocrinology and nutrition according to the clinical problem described.

### Statistical analysis

Categorical variables are presented as number (percentage), and continuous variables as mean ± standard deviation. Comparisons between groups were performed using the chi-square test or Fisher’s exact test for categorical variables, and the Student’s *t* test for continuous variables, as appropriate. To identify factors independently associated with referral inadequacy, univariable and multivariable logistic regression analyses were conducted, with referral adequacy (adequate vs inadequate) as the dependent variable. Given structural and organizational differences between referral pathways, separate regression models were built for primary care in-person referrals and hospital specialist in-person referrals.

Candidate independent variables included patient gender, age group, nosological group, and referral source. Age was analyzed as a categorical variable (16–40, 41–64, 65–79, and ≥80 years), using the 41–64-year group as the reference category due to its higher frequency. Gender was coded with female as the reference category. To avoid model overfitting and improve clinical interpretability, original diagnostic categories were aggregated into four clinically coherent nosological groups: thyroid disorders (reference), high-prevalence chronic metabolic disorders, high-complexity endocrine disorders, and other heterogeneous conditions. Referral source was categorized as health district reference center, local consulting clinic, or outside the hospital catchment area for primary care referrals, and as medical or surgical department for hospital referrals. Variables with clinical relevance and/or a univariable association with referral inadequacy (P <0.20) were considered for inclusion in multivariable models. Results are reported as odds ratios (ORs) with 95% confidence intervals (CIs). Model calibration was assessed using the Hosmer–Lemeshow goodness-of-fit test. Statistical significance was set at a two-sided P value <0.05. Analyses were performed using SPSS v.22.

## Results

### Study populations

A total of 1,106 referrals were analyzed during the study period, including 187 primary care e-consults, 447 primary care in-person referrals, and 472 hospital specialist in-person referrals (**Table 1**). Overall, women represented two-thirds of all referred patients (67.0%), although the sex distribution varied across referral pathways, with a higher proportion of female patients in primary care in-person referrals (74.0%) and a lower proportion in hospital specialist referrals (59.7%). The mean age of the cohort was 54.8 ± 18.2 years, with primary care e-consult patients being slightly older than those referred in-person from primary care. Thyroid dysfunction was the most frequent reason for consultation (24.3%), followed by diabetes (19.7%), while NTD and nutritional disorders showed marked variability depending on referral origin. Notably, NTD accounted for 18.8% of primary care in-person referrals but only 0.6% of hospital specialist referrals, and nutritional disorders were overwhelmingly concentrated in hospital referrals (16.7%).

**Table 1:** Classification of all e-consults and in-person referrals received according to their origin (primary care or hospital specialists).

	All referrals (n=1,106)	Primary care e-consults (A, n=187)	Primary care in-person referrals (B, n=447)	Hospital specialist in-person referrals (C, n=472)
Gender				
Female	741 (67.0)	128 (68.4)	331 (74.0)	282 (59.7)
Male	365 (33.0)	59 (31.6)	116 (26.0)	190 (40.3)
P (A vs B)			0.173	
P (B vs C)				**<0.001**
**Age, yr**	54.8 ± 18.2	56.1 ± 18.8	52.5 ± 17.8	56.4 ± 18.1
P (A vs B)			**0.028**	
P (B vs C)				**0.001**
Nosological group				
Thyroid dysfunction	269 (24.3)	56 (29.9)	90 (20.1)	123 (26.1)
NTD	117 (10.6)	30 (16.0)	84 (18.8)	3 (0.6)
Diabetes	218 (19.7)	32 (17.1)	96 (21.5)	90 (19.1)
Obesity	119 (10.8)	9 (4.8)	50 (11.2)	60 (12.7)
Dyslipidemia	31 (2.8)	11 (5.9)	13 (2.9)	7 (1.5)
Mineral metabolism	99 (9.0)	20 (10.7)	40 (8.9)	39 (8.3)
Adrenal disease	34 (3.1)	5 (2.7)	10 (2.2)	19 (4.0)
Nutrition	94 (8.5)	7 (3.7)	8 (1.8)	79 (16.7)
Other	125 (11.3)	17 (9.1)	56 (12.5)	52 (11.0)
P (A vs B)			**0.009**	
P (B vs C)				**<0.001**
Primary care source				
Health district reference center		142 (75.9)	282 (63.1)	
Local consulting clinic		45 (24.1)	105 (23.5)	
Out of hospital catchment area			60 (13.4)	
P (A vs B)			**<0.001**	
Hospital source				
Medical department				343 (72.7)
Surgical department				128 (27.1)
Other				1 (0.2)

Data are the mean ± SD for quantitative variables, and the number (percentage) for categorical variables.

NTD, nodular thyroid disease.

Values in bold represent statistically significant differences.

### Adequacy and inadequacy of referrals

**Figure 2:**
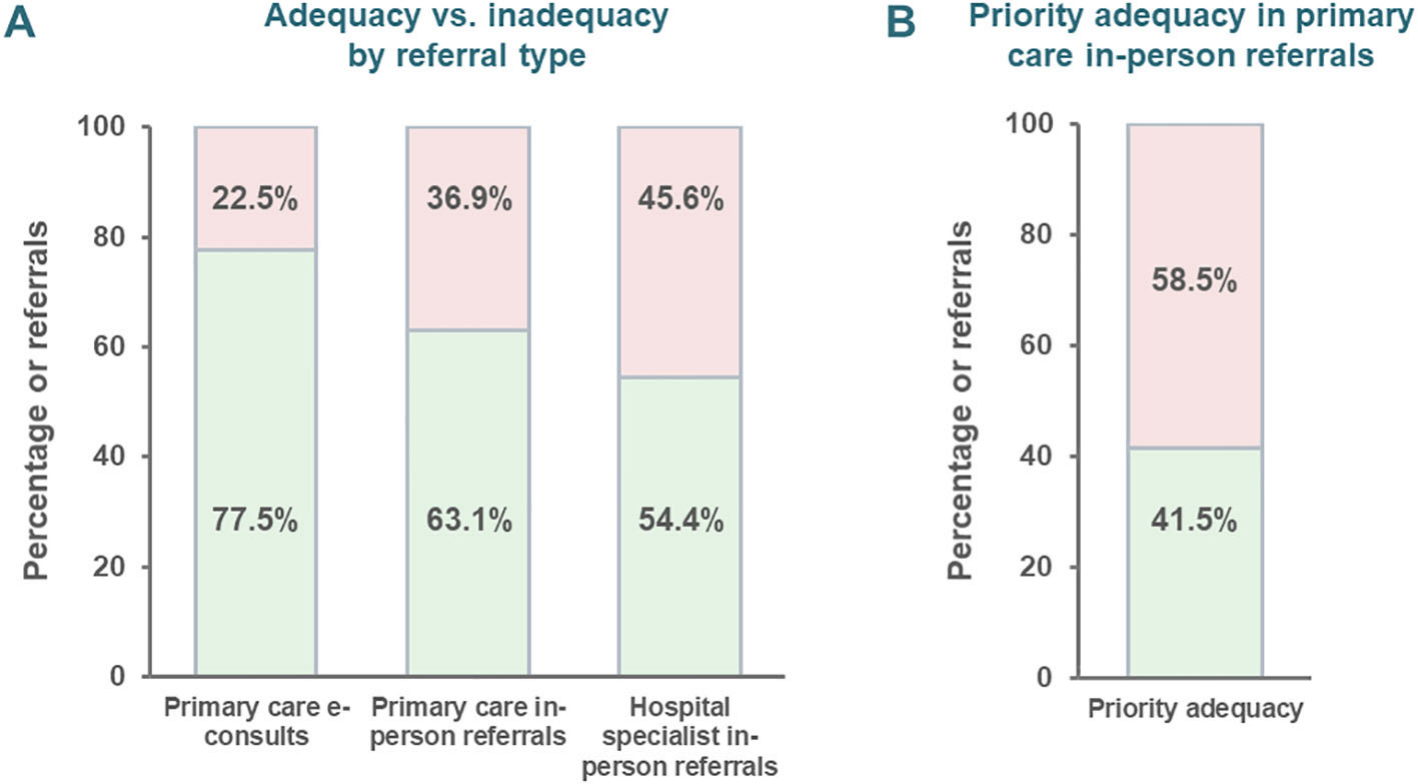
Adequacy and priority assignment by referral type. Panel **(A)** shows the proportion of adequate and inadequate referrals according to consultation modality, including primary care e-consults, primary care in-person referrals, and hospital specialist in-person referrals. Green bars represent adequate referrals and red bars represent inadequate referrals. Panel **(B)** depicts the adequacy of priority assignment among primary care in-person referrals classified as priority, with green bars indicating adequate priority assignment and red bars indicating inadequate priority assignment. Data are expressed as percentages.

Assessment of referral adequacy revealed substantial differences across consultation modalities (**Table 2**). Overall, 61.8% of all referrals were judged adequate, with primary care e-consults showing the highest adequacy rate (77.5%), followed by primary care in-person referrals (63.1%) and hospital specialist referrals (54.4%). Conversely, inadequacy was most frequent in hospital specialist referrals (45.6%) and least common in e-consults (22.5%) (**Figure 2A**).

**Table 2:** Adequacy of e-consults and in-person referrals received and cause of inadequacy by source of origin (primary care or hospital specialists).

	All	Primary care e-consults (A, n=187)	Primary care in-person referrals (B, n=447)	Hospital specialist in-person referrals (C, n=472)
Adequacy				
Adequate	684 (61.8)	145 (77.5)	282 (63.1)	257 (54.4)
Inadequate	422 (38.2)	42 (22.5)	165 (36.9)	215 (45.6)
P (A vs B)			**<0.001**	
P (B vs C)				**0.009**
Cause of inadequacy				
Clinical		6 (14.3)	165 (100)	86 (40.0)
Administrative		36 (85.7)	0 (0)	101 (47.0)
Both				28 (13.0)
P (A vs B)			**<0.001**	
P (B vs C)				**<0.001**

Data are the mean ± SD for quantitative variables, and the number (percentage) for categorical variables.

NTD, nodular thyroid disease.

Values in bold represent statistically significant differences.

When considering absolute numbers in addition to proportions, the distribution of inadequate referrals differed across pathways. Although e-consults showed the lowest proportion of inadequacy (22.5%), they accounted for a smaller number of total referrals (n = 187) and therefore contributed fewer inadequate cases (n = 42). In contrast, primary care and hospital in-person referrals, which represented larger volumes (n = 447 and n = 472, respectively), contributed substantially more to the total number of inadequate referrals (n = 165 and n = 215). These findings highlight that both relative proportions and absolute volumes should be considered when interpreting the contribution of each referral pathway to overall patterns of inadequacy.

Patterns of inadequacy differed notably between groups. Among e-consults, administrative inadequacy predominated (85.7%), largely due to inappropriate use of the platform for appointment requests or other non-clinical administrative purposes, while clinical inadequacy was uncommon (14.3%), most often driven by insufficient information or the absence of a relevant clinical question, as detailed in **Supplementary Table 3** (**Supplementary Material**). In primary care in-person referrals, all inadequacies were by definition clinical, predominantly related to insufficient clinical information or conditions that could be managed within primary care, as shown in **Supplementary Table 4** (**Supplementary Material**). Hospital specialist referrals exhibited a mixed pattern, with 40.0% classified as clinically inadequate—most commonly for issues manageable in primary care—and 47.0% classified as administratively inadequate, frequently because patients fell outside the hospital’s catchment area and the referral was unrelated to the specialist’s ongoing clinical process; an additional 13.0% presented both clinical and administrative deficiencies.

### Inadequacy according to patient characteristics, nosological group, and referral source

Analysis of inadequacy according to patient gender, age, nosological group, and referral source revealed distinct patterns across referral pathways (**Table 3**). In all three settings no significant association was observed between inadequacy and patient sex. Age, however, showed a significant relationship with inadequacy in both types of in-person referrals, with the highest rates consistently observed among patients aged 41–64 years in primary care and hospital referrals, whereas age was not significantly associated with inadequacy in e-consults. Nosological group was significantly associated with inadequacy across all referral modalities. Higher rates of inappropriateness were observed in referrals for obesity and thyroid dysfunction, particularly in in-person referrals, while lower inadequacy rates were noted for NTD and mineral metabolism disorders in selected settings. With regard to referral source, no significant differences were identified between health district reference centers and local consulting clinics for primary care e-consults or in-person referrals, although a non-significant trend toward higher inadequacy was observed in referrals originating outside the hospital catchment area. Among hospital specialist referrals, inadequacy rates did not differ significantly between medical and surgical departments. Given the number of comparisons performed, particularly across nosological groups, these results should be interpreted with caution as no formal correction for multiple testing was applied.

**Table 3:** Inadequacy of primary care e-consults and in-person referrals from primary care and hospital specialists according to patient gender, age, nosological group, and source of referral.

	Primary care						Hospital specialists		
	e-Consults			In-person referrals			In-person referrals		
	n/N	%	P	n/N	%	P	n/N	%	P
**Gender**			1.0			1.0			0.639
Female	29/128	22.7		122/331	36.9		131/282	46.5	
Male	13/59	22.0		43/116	37.1		84/190	44.2	
**Age, years**			0.434			**0.015**			**0.012**
16 a 40	7/41	17.1		32/116	27.6		39/107	36.4	
41 a 64	23/84	27.3		98/222	44.1		104/192	54.2	
65 a 79	7/42	16.7		28/87	32.2		55/127	43.3	
≥80	5/20	25.0		7/22	31.8		17/46	37.0	
**Nosological group**			**0.033**			**<0.001**			**<0.001**
Thyroid dysfunction	7/56	12.5		42/90	46.7		70/123	56.9	
NTD	5/30	16.7		38/84	45.2		0/3	0	
Diabetes	12/32	37.5		29/96	30.2		41/90	45.6	
Obesity	4/9	44.4		26/50	52.0		41/60	68.3	
Dyslipidemia	0/11	0		3/13	23.0		3/7	42.9	
Mineral metabolism	4/20	20.0		7/40	17.5		17/39	43.6	
Adrenal disease	2/5	40.0		1/10	10.0		6/19	31.3	
Nutrition	3/7	42.9		0/8	0		17/79	21.5	
Other	5/17	29.4		19/56	33.9		20/52	38.5	
**Source (primary care)**			1.0			0.130			
Health district reference center	32/142	22.5		95/282	33.7				
Local consulting clinic	10/45	22.2		42/105	40.0				
Out of hospital catchment area	—	—		28/60	46.7				
**Source (hospital specialists)**									0.382
Medical department							153/343	44.6	
Surgical department							61/128	47.7	
Other							0/1	0	

Data indicate the number of inadequate cases (n) out of the total number of individuals in each group (N), expressed as n/N and percentage (%).

Values in bold represent statistically significant differences.

### Inadequacy of priority assignment

Of the 447 in-person referrals originating from primary care, 207 (46.3%) were classified as priority referrals. Among these, only 86 (41.5%) met appropriate clinical criteria for priority scheduling, whereas 121 (58.5%) were considered inadequately prioritized (**Figure 2B**).

Compared with non-priority referrals, priority referrals were more frequently assigned to older patients, women, and cases related to thyroid dysfunction, NTD, and mineral metabolism disorders, and were more commonly generated by health district reference centers (**Supplementary Table 5**; **Supplementary Material**). Analysis restricted to priority referrals showed that inadequacy of priority was not significantly associated with patient gender, age, or primary care source, but differed markedly across nosological groups (**Table 4**). The highest proportions of inappropriate priority assignment were observed in referrals for obesity, dyslipidemia, diabetes, and mineral metabolism disorders, whereas lower rates were found for thyroid dysfunction and NTD. However, results by nosological group should be interpreted with caution, as some categories included a small number of cases (e.g., dyslipidemia, obesity, adrenal disease, and nutrition). Percentages derived from these small subgroups may be unstable and are presented for descriptive purposes only.

**Table 4:** Inadequacy of priority assignment in primary care in-person priority referrals according to patient characteristics, nosological group, and source.

	Priority referrals		
	n/N	%	P value
**Gender**			0.296
Female	94/166	56.6	
Male	27/41	65.9	
**Age, years**			0.506
16 a 40	26/40	65.0	
41 a 64	64/112	57.1	
65 a 79	20/39	51.3	
≥80	11/16	68.8	
**Nosological group**			**<0.001**
Thyroid dysfunction	21/55	38.2	
NTD	24/52	46.2	
Diabetes	29/37	78.4	
Obesity	7/8	87.5	
Dyslipidemia	2/2	100	
Mineral metabolism	21/27	77.8	
Adrenal disease	3/5	60.0	
Nutrition	2/3	66.7	
Other	12/18	66.7	
**Source (primary care)**			0.827
Health district reference center	92/155	59.4	
Local consulting clinic	24/44	54.5	
Outside catchment area	5/8	62.5	

Data indicate the number of referrals with inadequate priority (n) out of the total number of priority referrals in each group (N), expressed as n/N and percentage (%).

Values in bold represent statistically significant differences.

### Factors associated with inadequacy of in-person referrals

**Figure 3:**
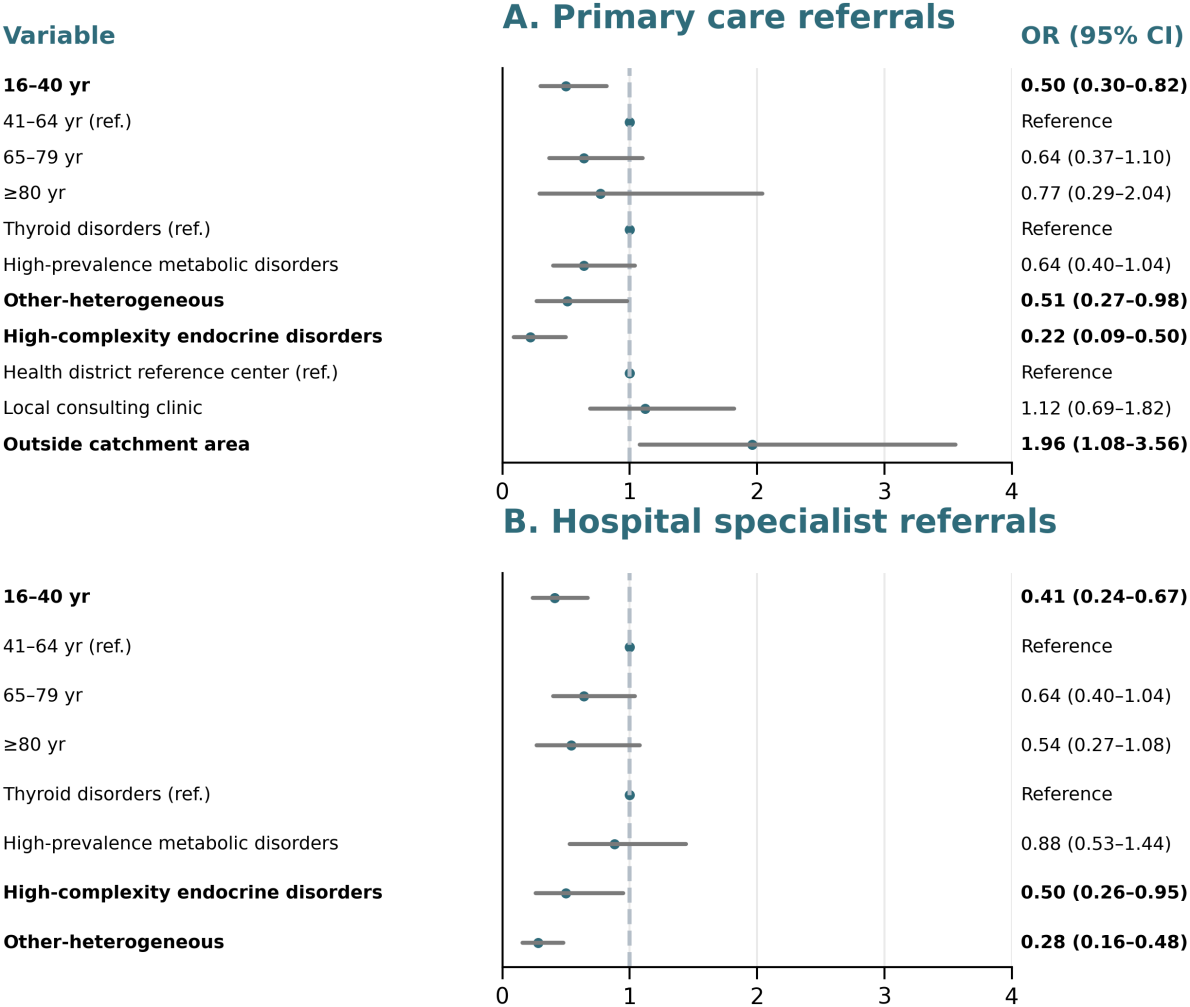
Forest plot of factors associated with referral inadequacy according to multivariable logistic regression models. Panel **(A)** shows results for primary care in-person referrals, and Panel **(B)** for hospital specialist referrals. Data are presented as odds ratios (ORs) with 95% confidence intervals. The vertical line indicates an OR of 1 (no association). Reference categories are indicated for each variable. *P < 0.05; **P < 0.01; ***P< 0.001.

Univariable and multivariable logistic regression analyses were performed separately for in-person referrals originating from primary care and hospital specialists to identify factors associated with referral inadequacy. In primary care referrals (**Table 5**), compared with the 41–64-year age group (reference category), both younger and older age groups showed lower odds of inadequacy. Regarding diagnostic categories, referrals for high-complexity endocrine disorders were consistently associated with a markedly lower likelihood of inadequacy compared with thyroid disorders, while referrals classified as other heterogeneous conditions also showed reduced odds of inadequacy. The aggregation of diagnostic categories in **Tables 5**, **6** was performed to facilitate analysis; however, given the heterogeneity and small sample sizes in some groups, these results should be interpreted with caution. Referral source outside the hospital catchment area was independently associated with higher odds of inadequacy. Gender was not significantly associated with referral adequacy in either univariable or multivariable analyses. Model calibration was adequate, as indicated by the Hosmer–Lemeshow goodness-of-fit test (χ² = 8.190, p = 0.415). The results of the multivariable logistic regression analyses are also presented graphically in **Figure 3A**.

**Table 5:** Results of univariable and multivariable logistic regression models to study the influence of several covariates as potential predictors of referral inadequacy of in-person referrals from primary care (n=447).

	Univariable			Multivariable		
	OR	95% CI	P	OR	95% CI	P
Gender						
Female (ref.)	1			1		
Male	1.01	0.65-1.56	0.968	1.13	0.70-1.83	0.627
Age group, years						
16-40	0.48	0.30-0.78	**0.003**	0.50	0.30-0.82	**0.006**
41-64 (ref.)	1			1		
65-79	0.60	0.36-1.01	0.055	0.64	0.37-1.10	0.105
≥80	0.59	0.23-1.51	0.276	0.77	0.29-2.04	0.599
Nosological group						
Thyroid disorders (ref.)	1			1		
High-prevalence metabolic disorders	0.68	0.44-1.05	0.079	0.64	0.40-1.04	0.070
High-complexity endocrine disorders	0.22	0.10-0.50	**<0.001**	0.22	0.09-0.50	**<0.001**
Other-heterogeneous	0.50	0.27-0.92	**0.025**	0.51	0.27-0.98	**0.042**
Source						
Health district reference center (ref.)	1			1		
Local consulting clinic	1.31	0.83-2.08	0.249	1.12	0.69-1.82	0.635
Outside catchment area	1.72	0.98-3.03	0.059	1.96	1.08-3.56	**0.027**

OR odds ratio, CI confidence interval. OR >1 indicate higher odds of referral inadequacy.

The 9 nosological groups are distributed in four groups: thyroid disorders (thyroid dysfunction and nodular thyroid disease), high-prevalence metabolic disorders (diabetes, obesity and dyslipidemia), high-complexity endocrine disorders (mineral metabolism and adrenal disease), and other-heterogeneous (nutrition and others).

Values in bold represent statistically significant differences.

**Table 6:** Results of univariable and multivariable logistic regression models to study the influence of several covariates as potential predictors of referral inadequacy of in-person referrals from hospital specialists (n=471*).

	Univariable			Multivariable		
	OR	95% CI	P	OR	95% CI	P
Gender						
Female (ref.)	1			1		
Male	0.91	0.63-1.32	0.631	0.94	0.62-1.42	0.768
Age group, years						
16-40	0.49	0.30-0.79	**0.004**	0.41	0.24-0.67	**0.001**
41-64 (ref.)	1			1		
65-79	0.65	0.41-1.02	0.058	0.64	0.40-1.04	0.070
≥80	0.50	0.26-0.96	**0.038**	0.54	0.27-1.08	0.082
Nosological group						
Thyroid disorders (ref.)	1			1		
High-prevalence metabolic disorders	0.94	0.59-1.51	0.812	0.88	0.53-1.44	0.603
High-complexity endocrine disorders	0.53	0.28-0.99	**0.046**	0.50	0.26-0.95	**0.034**
Other-heterogeneous	0.32	0.19-0.53	**<0.001**	0.28	0.16-0.48	**<0.001**
Source						
Medical department (ref.)	1			—		
Surgical department	1.13	0.75-1.70	0.554	—		

OR odds ratio, CI confidence interval. OR >1 indicate higher odds of referral inadequacy.

The 9 nosological groups are distributed in four groups: thyroid disorders (thyroid dysfunction and nodular thyroid disease), high-prevalence metabolic disorders (diabetes, obesity and dyslipidemia), high-complexity endocrine disorders (mineral metabolism and adrenal disease), and other-heterogeneous (nutrition and others).

*A patient who did not come from a medical or surgical service is excluded.

Values in bold represent statistically significant differences.

In hospital specialist referrals (**Table 6**; **Figure 3B**), similar patterns were observed. Compared with the 41–64-year age group, both younger and older age groups showed lower odds of inadequacy, and referrals for high-complexity endocrine disorders and other heterogeneous conditions were significantly less likely to be inadequate than those for thyroid disorders. Neither patient sex nor referral source (medical vs surgical departments) was significantly associated with referral adequacy. Model calibration was also adequate (Hosmer–Lemeshow test: χ² = 4.907, p = 0.767).

## Discussion

The present study provides a comprehensive evaluation of referral adequacy to an endocrinology service across different consultation modalities and highlights several clinically and organizationally relevant findings. First, referral inadequacy was common, affecting more than one third of all consultations, with substantial variability according to the type of referral. Second, primary care e-consults showed the highest adequacy rates, supporting their role as an effective and efficient tool for specialist support when appropriately used. Third, hospital specialist in-person referrals exhibited the highest proportion of inadequacy, reflecting persistent clinical and administrative misalignment between specialties. While hospital referrals showed a higher proportion of inadequacy compared with other pathways, their overall contribution should be interpreted in the context of their larger volume. When absolute numbers are considered, in-person referrals from both primary care and hospital settings account for the majority of inadequate cases. These results suggest that improvement efforts should consider both the proportion of inadequate referrals within each pathway and the overall volume of referrals, as both dimensions contribute to the total burden (20). Finally, more than half of primary care referrals labeled as priority did not meet appropriate criteria for preferential scheduling, revealing significant shortcomings in prioritization processes (21). Together, these findings underscore the complexity of referral pathways to endocrinology services and point to multiple, potentially modifiable targets for improving referral quality, optimizing resource utilization, and ensuring timely access to specialist care for patients who are most likely to benefit. Importantly, these findings are not only statistically significant but also clinically relevant, as they identify specific referral patterns and organizational contexts in which interventions may have the greatest impact.

### Referral adequacy

Referral inadequacy to endocrinology services was frequent in our cohort, affecting more than one third of all consultations, a finding that is broadly consistent with previous reports across different healthcare systems. Studies conducted in primary and specialty care settings have consistently shown that between 20% and 50% of referrals to specialist services may be considered inappropriate, depending on the specialty, the definition applied, and the organizational context (14–17). Our overall inadequacy rate falls within this range and reinforces the notion that referral quality remains a persistent challenge in the management of chronic, high-prevalence conditions such as endocrine and metabolic disorders. Compared with earlier studies focused exclusively on in-person referrals, our analysis provides a more nuanced picture by simultaneously evaluating e-consults and hospital-based referrals, revealing marked heterogeneity across referral pathways. While some previous reports have attributed high inadequacy rates primarily to deficiencies in primary care referrals (18), our findings highlight that hospital specialist referrals may contribute equally—or even more substantially—to overall inefficiency, particularly when administrative and organizational factors are considered. It should be noted here that a volume-weighted perspective is essential to accurately identify priority areas for intervention within the referral system.

### e-consults

The binary classification of adequacy used in this study should be interpreted within the specific conceptual framework adopted. In the case of e-consults, adequacy was defined in terms of the quality and relevance of the clinical query, rather than the appropriateness of subsequent patient management. As such, different clinical scenarios leading to in-person care or no further intervention were not distinguished within the inadequacy category. This approach reflects the intended function of e-consults in our setting but may differ from other models where triage or care pathways are explicitly incorporated. Furthermore, our findings should be interpreted within a setting in which the e-consultation system is well established, widely used, and not influenced by financial incentives, suggesting that observed patterns are unlikely to reflect early implementation effects.

Primary care e-consults demonstrated the highest adequacy in our study, which aligns closely with recent literature highlighting the efficiency and clinical value of such tools in endocrinology. A comprehensive scoping review of endocrine e-consults reported consistently positive findings on feasibility, timeliness, and acceptability, noting that when implemented properly, e-consults were at least equivalent to face-to-face referrals in improving glycemic control and facilitating specialist-recommended interventions (11). Furthermore, in the Champlain BASE e-consult system, 62% of primary care providers reported receiving new or additional management advice, and 44% of face-to-face appointments were avoided, with over 95% rating the tool highly valuable (22). A recent systematic review focused on diabetes care also confirmed that e-consults enhance access to specialist expertise, reduce costs, shorten wait times, and improve provider education, although direct impacts on HbA1c levels were mixed (23). Our findings reinforce these observations, demonstrating a 77.5% adequacy rate—significantly outperforming in-person referrals. However, unlike some studies, we found that administrative misuse remains common, underscoring the need for targeted implementation strategies and institutional protocols to maintain high-quality utilization of e-consults.

### Hospital referrals

Hospital specialist in-person referrals exhibited the highest rate of inadequacy in our study, a finding that both aligns with and expands upon previous literature examining referral quality within secondary and tertiary care. While much of the existing research has traditionally focused on inappropriate referrals from primary care, several studies have highlighted that referrals originating from hospital specialists are not exempt from inefficiencies and may contribute substantially to inappropriate specialist utilization (16, 17, 24). Similar to our results, prior analyses have identified that a significant proportion of hospital-based referrals are driven by organizational or administrative factors rather than by unmet clinical needs, particularly when patients fall outside institutional catchment areas or when referrals are not directly related to the clinical process managed by the requesting service (15). Our findings extend this evidence by demonstrating that hospital referrals not only showed the highest overall inadequacy rate but also a mixed pattern of clinical and administrative deficiencies, underscoring the complexity of interspecialty referral pathways. In contrast to some reports suggesting that referrals between specialists are inherently more appropriate due to shared clinical expertise (9), our data indicate that misalignment of roles, scope of practice, and organizational boundaries can undermine referral quality even within hospital settings. These discrepancies likely reflect local structural factors and emphasize the need for clearer interdepartmental referral criteria and accountability mechanisms to improve efficiency within specialized care.

### Priority of referrals

A particularly relevant finding of our study was that more than half of primary care in-person referrals labeled as priority did not meet appropriate criteria for preferential scheduling. This observation is consistent with previous reports indicating substantial variability and frequent overuse of urgent or priority referral pathways in outpatient specialty care (17, 18). Prior studies have shown that prioritization decisions in primary care are often influenced by factors beyond objective clinical urgency, including perceived patient expectations, diagnostic uncertainty, and limited access to diagnostic resources, which may lead to precautionary over-prioritization (15, 16, 25, 26). Similar to our findings, other authors have reported that high-prevalence chronic conditions—such as diabetes, obesity, and dyslipidemia—are disproportionately represented among inappropriately prioritized referrals, despite often being manageable through standard referral pathways (18, 27). In contrast, lower rates of inappropriate prioritization have been described for conditions traditionally perceived as requiring specialized or time-sensitive evaluation, such as NTD, which aligns with our results (28). Nevertheless, although in our study higher proportions of inadequate prioritization were observed in certain diagnostic groups, these findings should be interpreted cautiously due to the small number of cases in several categories. Therefore, these results are exploratory and should not be used to infer definitive patterns of inappropriate prioritization across specific conditions. Collectively, these findings have clear practical implications, as inappropriate use of priority pathways may directly affect waiting times and access for patients with genuinely urgent endocrine conditions.

### Factors influencing referral adequacy

Beyond the main findings, our study provides additional insights into patient- and system-level factors influencing referral adequacy. The association between age and adequacy did not follow a linear pattern. The 41–64-year group, used as the reference category, showed the highest proportion of inadequacy, while both younger and older patients had lower odds. This finding suggests that the relationship between age and referral adequacy is more complex than initially assumed. A possible explanation is that the 41–64-year group represents a clinically heterogeneous population, in whom diagnostic uncertainty or variability in referral thresholds may be greater. In contrast, younger patients may more frequently present with well-defined conditions, while older patients may already be under specialist follow-up or present with clearer referral indications (16, 17, 29, 30). However, these interpretations are speculative and were not directly assessed in this study. Therefore, these findings should be considered exploratory and hypothesis-generating.

While some associations reached statistical significance, not all may have direct clinical implications. In contrast, factors related to diagnostic category and organizational context appear to have more actionable relevance for improving referral practices. In fact, diagnostic category was another strong and consistent predictor of referral adequacy. Referrals for high-complexity endocrine disorders were significantly less likely to be inadequate than those for high-prevalence chronic metabolic or thyroid conditions, a finding that aligns with prior literature indicating that rarer or more specialized conditions tend to prompt more careful referral practices (11, 15, 31, 32). In contrast, high-prevalence conditions such as diabetes, obesity, and thyroid dysfunction—frequently managed in primary care—have repeatedly been associated with higher variability in referral adequacy, particularly when clear referral thresholds are lacking (33). The interpretation of results across diagnostic categories requires consideration of how these groups were defined. The grouping of multiple diagnostic categories into broader nosological groups may have influenced the observed associations, including which comparisons reached statistical significance. While this approach was necessary to reduce fragmentation and allow analysis given the limited number of cases in several categories, it may obscure clinically relevant differences between conditions. Furthermore, alternative grouping strategies could yield different results, particularly in the context of small subgroup sizes. In addition, multiple statistical comparisons were performed without formal adjustment for multiplicity, which may increase the risk of type I error. Therefore, associations observed across diagnostic groups should be interpreted as exploratory.

Organizational factors also played a relevant role. Referrals originating outside the hospital catchment area were independently associated with higher inadequacy in primary care referrals, highlighting how administrative boundaries and structural misalignment between care levels can negatively affect referral quality. This finding is consistent with reports emphasizing the impact of health system organization, referral circuits, and accountability frameworks on specialist utilization (18, 27). Conversely, patient sex and referring specialty (medical vs surgical) were not independently associated with referral adequacy, suggesting that inappropriate referrals are driven more by clinical context and system organization than by demographic factors or professional background (34). This reinforces the view that improving referral quality requires system-level interventions rather than focusing solely on individual clinician characteristics.

### Clinical implications

From a clinical and organizational perspective, our findings have several important implications for both frontline clinicians and healthcare system managers. For clinicians in primary care and hospital settings, the high prevalence of referral inadequacy reinforces the need for clearer, diagnosis-specific referral criteria and improved decision-support tools to guide appropriate specialist involvement, particularly for high-prevalence endocrine conditions (15, 17). The consistently higher adequacy observed in e-consults supports previous evidence indicating that structured asynchronous communication between primary care and specialists can enhance referral quality, reduce unnecessary in-person visits, and improve access to specialist advice when appropriately implemented (10–12). From a management and policy perspective, the substantial inefficiencies identified in hospital specialist referrals and in priority assignment underscore the importance of reviewing referral circuits, clarifying interdepartmental responsibilities, and standardizing prioritization frameworks to align clinical urgency with access to specialist care (18). Moreover, the association between organizational factors—such as referrals originating outside the hospital catchment area—and higher inadequacy highlights how structural and administrative misalignment across care networks can negatively affect referral quality (16, 17). Collectively, these findings support a multifaceted strategy combining clinician education, optimized use of e-consult platforms, structured referral and prioritization criteria, and organizational reforms, aimed at improving efficiency, reducing unwarranted variability, and ensuring timely access to endocrinology services for patients most likely to benefit.

The findings of this study should be interpreted within the context of a process-based evaluation. The assessment of adequacy does not capture downstream effects such as additional primary care visits, diagnostic testing, delayed referrals, or clinical outcomes. Therefore, a classification of inadequacy should not be interpreted as evidence of inefficiency at the healthcare system level. These downstream trajectories were not assessed in this study and warrant further investigation. In addition, structural aspects of the healthcare system may influence the observed patterns of adequacy (35). In our setting, primary care and hospital care do not share a fully integrated clinical record system, which may contribute to cases of insufficient clinical information. This suggests that some instances of inadequacy may be related to system-level constraints rather than solely to the referring clinician.

Referral adequacy should also be interpreted in the context of clinical uncertainty. Requests for specialist input may appropriately arise in borderline or complex situations where predefined criteria do not fully apply. Therefore, referral criteria should not be viewed as rigid thresholds, but as context-dependent tools that support, rather than replace, clinical judgment. In this regard, efforts to improve referral adequacy should balance standardization with professional autonomy and include collaborative strategies such as enhanced communication between care levels and ongoing education, rather than relying exclusively on stricter referral criteria (36).

### Potential areas for improvement

Beyond describing patterns of referral adequacy, our findings suggest several areas for potential improvement in the use of e-consults and referral pathways. First, a substantial proportion of clinically inadequate e-consults was related to insufficient clinical information, indicating that structured templates or minimum data requirements could enhance the quality of queries and facilitate specialist responses (37). Second, the high proportion of administratively inappropriate e-consults suggests a need to reinforce the intended clinical purpose of this tool, potentially through targeted training or clearer institutional guidance (38). Third, given that the largest absolute number of inadequate referrals occurred in in-person pathways, particularly hospital referrals, improvement strategies should not focus exclusively on e-consults but rather address referral practices across the entire system. Finally, some causes of inadequacy appear to be related to structural factors, such as the lack of shared clinical records between primary and hospital care, highlighting potential areas for system-level intervention (20).

### Strengths and limitations

This study has several notable strengths. First, it provides a comprehensive and simultaneous evaluation of all referral modalities to an endocrinology service, including primary care e-consults, primary care in-person referrals, and hospital specialist referrals, allowing direct comparison across referral pathways within the same organizational context. Second, the prospective design and inclusion of all consecutive referrals over a defined period minimize selection bias and enhance the internal validity of the findings. Third, the relatively large sample size enabled detailed subgroup analyses and multivariable modeling, strengthening the robustness of the associations identified. Finally, the use of predefined, consensus-based criteria for assessing referral adequacy and priority assignment reflects real-world clinical practice and increases the clinical relevance of the results.

Several limitations should also be acknowledged. First, clinical adequacy criteria were developed and validated internally within the same department that later performed the assessment, without external independent validation. Second, adequacy assessment was conducted by multiple evaluators without formal inter-rater agreement analysis and without blinded or fully independent evaluation, which may limit reproducibility. Third, the absence of an external assessment panel, including primary care physicians, may introduce a degree of specialty-related perspective in defining what constitutes an appropriate referral. Nevertheless, several measures were implemented to enhance internal consistency, including the use of explicitly predefined criteria, their formal approval by all department members, and consensus-based resolution of uncertain cases.

An additional potential limitation relates to the fact that referral adequacy was assessed by the same department receiving the referrals, which may raise concern regarding structural bias. However, adequacy classification in this study was performed retrospectively and had no impact on patient management, waiting lists, or workload distribution. Furthermore, clinicians responsible for patient care were blinded to the study assessment, and evaluators had no incentive to classify referrals in a particular direction. The primary aim was to obtain real-world data to inform future quality improvement initiatives.

Additionally, the analysis was conducted at the level of individual referrals rather than patients and therefore did not account for multiple referrals from the same patient or for repeated consultations addressing the same clinical issue. Finally, additional factors that may influence referral adequacy, such as the healthcare setting (e.g., rural versus urban), patient socioeconomic characteristics, and referring physician experience, were not available in our dataset and could not be analyzed. These variables may contribute to variability in referral patterns and should be considered in future studies.

Another limitation of this study is that we did not assess the quality of the specialist’s response to the e-consultation. Our analysis focused exclusively on the adequacy of the incoming clinical query, in line with the primary objective of evaluating the adequacy of e-consults use from primary care. Future research should address this aspect by incorporating measures of response quality and its impact on clinical decision-making and patient outcomes. We should mention here that, in cases of specialist-to-specialist referrals, we evaluated only the adequacy of the referral to our department, without considering whether earlier routing decisions within the healthcare system were appropriate. Consequently, some cases classified as inadequate may reflect upstream referral processes rather than the decision of the referring specialist at the final step.

This was a single-center study conducted within a specific healthcare system, which may limit the generalizability of the findings to other settings with different organizational structures or referral pathways. Additionally, the study was not designed to explore the underlying causes of age-related differences in referral adequacy, limiting causal interpretation of these findings. The study focused on referral processes and did not evaluate downstream clinical outcomes, patient satisfaction, or economic impact, which may further inform the consequences of referral inadequacy. Finally, e-consult adequacy was assessed within the context of locally defined institutional rules, which may differ from those applied in other health systems.

## Conclusions

In conclusion, referral inadequacy to endocrinology services remains a common and multifactorial problem, influenced by consultation modality, diagnostic category, prioritization practices, and organizational context. Our findings demonstrate that appropriately implemented e-consults can achieve high levels of adequacy, while hospital specialist referrals and priority assignment represent critical areas for improvement. This study provides a detailed description of referral adequacy across different pathways and identifies specific areas for improvement, including the quality of clinical information in e-consults, the reduction of administrative misuse, and the need to address high-volume referral pathways. These findings may inform targeted strategies to optimize the use of e-consultation within integrated care systems. Future research should assess the impact of standardized referral criteria, decision-support tools, and expanded e-consult use on clinical outcomes, patient satisfaction, and healthcare resource utilization, as well as explore the generalizability of these findings across different healthcare systems.

## Data Availability

The raw data supporting the conclusions of this article will be made available by the authors, without undue reservation.
