# Supplementary material for: Adequacy of electronic and in-person referrals to endocrinology: a prospective study of primary care and hospital consultations
Source: Front Endocrinol (Lausanne). 2026 Jul 24;17:1864526. doi: 10.3389/fendo.2026.1864526 (PMC13447361; doi:10.3389/fendo.2026.1864526)
Supplement: Supplementary file 1 [file Table1.docx]

**Adequacy of Electronic and In‑Person Referrals to Endocrinology: A Prospective Study of Primary Care and Hospital Consultations**

**SUPPLEMENTARY MATERIAL**

**Table S1.** Criteria for assessing clinical and administrative inadequacy in e-consults and in-person referrals

| **Type** | **Source** | **Clinical inadequacy** | **Administrative inadequacy** |
| --- | --- | --- | --- |
| e-Consults | Primary care | 1. Insufficient clinical information to provide a reasoned specialist recommendation 2. Non‑applicable e‑consult: absence of a clearly formulated clinical question or query outside the scope of Endocrinology and Nutrition | 1. Appointment request or appointment‑related management 2. Other non‑clinical administrative procedures |
| In-person referrals | Primary care | 1. Insufficient clinical information 2. Condition manageable within primary care 3. Condition not related to Endocrinology and Nutrition | Not aplicable |
| In-person referrals | Hospital specialists | 1. Insufficient clinical information 2. Condition manageable within primary care 3. Condition not related to Endocrinology and Nutrition | 1. Referral from outside the hospital catchment area and unrelated to the clinical process managed by the referring specialty 2. Other administrative causes |

**Table S2.** Criteria for adequate in-person referrals classified by nosological groups

| **Nosologic group** | **Referral criteria** |
| --- | --- |
| **Thyroid dysfunction** | - Difficult‑to‑control primary hypothyroidism - Central hypothyroidism - Pregnant women or women planning pregnancy with poorly controlled hypothyroidism - Subclinical hyperthyroidism confirmed by two determinations of thyrotropin (TSH < 0.3 mIU/L) - Overt hyperthyroidism - Central hyperthyroidism - Hyperthyroidism in pregnant women or women planning pregnancy |
|  |  |
| **Nodular thyroid disease** | - Thyroid nodule or cervical mass with rapid growth - Thyroid nodule associated with low TSH levels - Thyroid nodule with normal or elevated TSH and any of the following ultrasound criteria:   - TI‑RADS 5 (high suspicion)   - TI‑RADS 4 (intermediate suspicion) > 1.5 cm   - TI‑RADS 3 (low suspicion) > 2.5 cm   - TI‑RADS 1 or 2 (benign) > 3 cm - Thyroid nodule > 1 cm detected on PET‑CT |
|  |  |
| **Diabetes** | - Type 1 diabetes mellitus - Suspected monogenic diabetes (MODY), latent autoimmune diabetes in adults (LADA), or pancreatogenic diabetes (type 3c) - Women with diabetes planning pregnancy - Poorly controlled type 2 diabetes (HbA1c > 7.5% in patients < 65 years or > 8.0% in patients ≥ 65 years) - Diabetes with neurological or macrovascular complications - Pregnant women with pre‑existing diabetes - Gestational diabetes mellitus |
|  |  |
| **Obesity** | - Suspected syndromic obesity - Suspected secondary obesity - Body mass index (BMI) > 40 kg/m² - BMI > 35 kg/m² with one or more obesity‑related comorbidities, including:   - Poorly controlled type 2 diabetes despite treatment with SGLT2 inhibitors or GLP‑1 receptor agonists   - Obstructive sleep apnea with intolerance to CPAP therapy   - Hypertension with blood pressure > 140/90 mmHg despite ≥ 3 antihypertensive agents   - Severe osteoarthritis of weight‑bearing joints in patients < 60 years - BMI > 35 kg/m² in patients in whom weight loss is a clinical priority (e.g., transplant candidates, infertility) |
|  |  |
| **Dyslidepemia** | - Severe hypercholesterolemia (LDL‑cholesterol ≥ 190 mg/dL) - Severe hypertriglyceridemia (triglycerides ≥ 500 mg/dL or ≥ 300 mg/dL persistent after dietary intervention) - Suspected familial dyslipidemia - Failure to achieve lipid targets despite maximal statin therapy with good adherence - Statin intolerance or adverse effects to lipid‑lowering therapies - Complex mixed dyslipidemia - High or very high cardiovascular risk with inadequate lipid control |
|  |  |
| **Mineral metabolism** | - Abnormal serum calcium or phosphorus levels - Suspected primary hyperparathyroidism - Suspected hypoparathyroidism - Suspected metabolic bone diseaseg - Severe vitamin D deficiency |
| **Adrenal disease** | - Suspected adrenal insufficiency - Suspected hypercortisolism (Cushing syndrome) - Suspected primary hyperaldosteronism - Suspected pheochromocytoma - Adrenal incidentaloma or adrenal mass of any type |
|  |  |
| **Nutrition** | - Patients with moderate or severe malnutrition (unintentional weight loss ≥ 10% within < 6 months) due to:   - Reduced intake or impaired transit due to oropharyngeal, neurological or mechanical dysphagia   - Reduced absorption due to malabsorption syndromes or enterocutaneous fistulas   - Increased or special requirements: elective surgery or transplantation, cancer‑related cachexia, chronic diseases associated with increased metabolic demand, and postoperative gastrointestinal conditions - Patients with eating disorders - Patients requiring home artificial nutrition: enteral nutrition or parenteral nutrition |

Abbreviations: BMI, body mass index; CPAP, continuous positive airway pressure; HbA1c, glycated hemoglobin; PET‑CT, positron emission tomography–computed tomography; SGLT2, sodium–glucose cotransporter‑2; GLP‑1, glucagon‑like peptide‑1.

**Table S3.** Reasons for clinical and administrative inadequacy in primary care e-consults

| **Type of inadequacy** | **Primary care e-consults**  **(n=42)** |
| --- | --- |
| **Clinical inadequacy** | 6 (14.3) |
| Insufficient clinical information | 3 (50.0) |
| No clinical question posed or question outside the scope of endocrinology | 3 (50.0) |
|  |  |
| **Administrative inadequacy** | 36 (85.7) |
| Appointment request | 35 (97.2) |
| Other administrative procedures | 1 (2.8) |

Data are the number (percentage).

**Table S4.** Reasons for clinical and administrative inadequacy in in-person referrals from primary care and hospital specialists

| **Type of inadequacy** | **Primary care in-person referrals (n=165)** | **Hospital specialist in-person referrals (n=215)** |
| --- | --- | --- |
| **Clinical inadequacy** | 165 | 114 (53.0)* |
| Insufficient clinical information | 68 (41.2) | 20 (17.5) |
| Condition manageable in primary care | 89 (53.9) | 92 (80.7) |
| Condition not related to endocrinology | 8 (4.8) | 2 (1.8) |
|  |  |  |
|  |  |  |
| **Administrative inadequacy** | 0 | 129 (60.0)* |
| Patient outside catchment are and referral unrelated to ongoing specialist process |  | 113 (87.6) |
| Other administrative procedures |  | 16 (12.4) |

Data are the number (percentage).

*In the group of hospital specialist in-person referrals there are 28 cases of inadequacy due to both clinical and administrative reasons.

**Table S5.** Comparison of primary care in‑person referrals according to assigned priority

|  | **Priority referrals (n=207)** | **Non-priority referrals (n=240)** | **P value** |
| --- | --- | --- | --- |
| **Gender** |  |  | **0.007** |
| Female | 166 (80.2) | 165 (68.8) |  |
| Male | 41 (19.8) | 75 (31.3) |  |
|  |  |  |  |
| **Age, yr** | 55.4±17.0 | 50.1±18.2 | **0.002** |
|  |  |  |  |
| **Nosological group** |  |  | **<0.001** |
| Thyroid dysfunction | 55 (26.6) | 35 (14.6) |  |
| NTD | 52 (25.1) | 32 (13.3) |  |
| Diabetes | 37 (17.9) | 59 (24.6) |  |
| Obesity | 8 (3.9) | 42 (17.5) |  |
| Dyslipidemia | 2 (1.0) | 11 (4.6) |  |
| Mineral metabolism | 27 (13.0) | 13 (5.4) |  |
| Adrenal disease | 5 (2.4) | 5 (2.1) |  |
| Nutrition | 3 (1.4) | 5 (2.1) |  |
| Other | 18 (8.7) | 38 (15.8) |  |
|  |  |  |  |
| **Primary care source** |  |  | **<0.001** |
| Health district reference center | 155 (74.9) | 127 (52.9) |  |
| Local consulting clinic | 44 (21.3) | 61 (25.4) |  |
| Out of hospital catchment area | 8 (3.9) | 52 (21.7) |  |

Data are presented as mean ± SD for quantitative variables and number (percentage) for categorical variables.

Abbreviations: NTD, nodular thyroid disease
